# Bone marrow embolism: should it result from traumatic bone lesions? A histopathological human autopsy study

**DOI:** 10.1007/s12024-023-00609-2

**Published:** 2023-05-03

**Authors:** Maha Farid, Esraa Zohny, Alaa Ismail, Mariem Ateya, Ahmed Abdel-Razek, Nermien Hamed, Alaa Elmarakby, Arwa Hassanin, Ahmed Ismail, Omar Mansour, Hossam Roshdy, Yehia Ahmed, Mariam Ismail, Hebat Allah A. Amin

**Affiliations:** 1 Department of Forensic Medicine and Clinical Toxicology, Faculty of Medicine, Helwan University, Cairo, Egypt; 2 Faculty of Medicine, Helwan University, Cairo, Egypt; 3 Department of Pathology, Faculty of Medicine, Helwan University, Cairo, Egypt

**Keywords:** Bone marrow embolism, Fat embolism, Shock lung, Megakaryocyte embolism, Traumatic death

## Abstract

Bone marrow embolism (BME) is likely a consequence of fractures in which pulmonary vessels are the most affected. However, some cases of BME were reported in the absence of trauma. Thus, a traumatic injury might not be necessary for developing BME. This study discusses BME cases in patients without signs of fractures or blunt trauma. The discussion addresses various possible mechanisms for the appearance of BME. Options include cancer in which bone marrow metastasis is a suggestive cause. Another proposal is the chemical theory where bone marrow fats are released via lipoprotein lipase in a pro-inflammatory state, resulting in vascular/pulmonary obstruction. Other cases discussed in this study are hypovolemic shock and drug-abuse related BME. All autopsy cases with BME were included regardless of the cause of death for a period of 2 years. Autopsies involved complete dissection with the macroscopic evaluation of the affected organs, including the heart, lungs, and brain. Tissues were also prepared for microscopic examination. Of the 11 cases, eight showed non-traumatic BME (72%). These findings conflict with theories in the literature that BME most commonly occurs after fractures or trauma. One of the eight cases exhibited mucinous carcinoma; one is presented with hepatocellular carcinoma; and two cases showed severe congestion. Lastly, one case was found to be associated with each of the following conditions: liposuction, drug abuse, pulmonary hypertension, and heart failure. Each case suggests a different pathophysiology for developing BME, yet the exact mechanisms are not fully understood. Further study of non-traumatic associated BME is recommended.

## Introduction

Non-thrombotic pulmonary embolism is a less common cause of morbidity and mortality when compared to thrombotic pulmonary embolism. The latter refers to embolization of the pulmonary circulation mainly containing non-thrombotic elements, e.g., bacteria, foreign materials, and marrow elements. Bone marrow embolism (BME) is rare and generally understood to occur after trauma to the bones containing red marrow. The embolus is mainly composed of bone marrow elements including  marrow adipocytes. Frequently, small-sized pulmonary arteries are the most affected. Separate cases of BME however, have been observed in the coronary arteries [1, 2].

The first evidence of BME in man and animals was reported by Lubarsch [3] and Lengemann [4]. The suggested predisposing factors were (1) disturbances of the bone marrow, characterized mainly by decreased cohesiveness of cellular elements, and (2) bone contusion even without accompanying fractures [5]. Maximow [6] and others [7–10] reported BME in animals and suspected that trauma, and even contusion, was the underlying cause. Additional human cases were reported by Sotti [11] and others [12–15]. Rappaport et al. suggested that BME may occur after fractures of the red marrow-containing bones [5]. A fracture may occur either internally, resulting from violent muscular contractions after convulsions, or externally [5]. Subsequently, most cases of BME have relied on fractures for determining the origin. Even in circumstances where facture was not apparent, death was assumed to be due to existing, but undetected, fractures [5]. Trauma-related BME, as proposed by Gauss, is caused by torn medullary veins, where the pressure of the marrow increasingly forces it into the venous system [16].

Several trauma-inducing factors have been proposed. Grandi et al. analyzed 53 cases, where in 31 cases, emboli were attributed to cardiac massage and in an individual case to an accident. No clear etiology was identified for the remaining 21 cases [17]. Likewise, Buchanan and Mason [18] and others [2, 19, 20] attributed the occurrence of BME to resuscitative measures in cases of natural death. Arai indicated a significant correlation between the size of emboli and the extent of bone destruction, whereas a non-significant correlation between the number of emboli and the magnitude of destruction [21]. Moreover, Blumenthal and Saayman [22] reported two cases of BME in electrocution; one of the subjects showed a skeletal injury from high-voltage exposure, and the other case, involving domestic current, displayed no evidence of skeletal injury. Iatrogenic BME has been reported in the case of Gleason and Aufderheide [23], who suggested that the marrow of tuberculous vertebrae accidentally entered the circulation under compression during cystoscopy. BME is noted as a complication of multiple myeloma [24] and sickle cell anemia [25, 26] and a consequence of costal and sternal fractures in the course of malignant neoplasms and shock [27].

Another significant BME case reported BME in clinically suspected dengue shock syndrome [28], raising the controversial question of whether traumatic lesions are a necessary prequel to BME. Yet, evidence of BME occurring in non-traumatic cases is limited. Recently, a general agreement has established that BME is associated with skeletal injury [29], reflecting a paucity of evidence on non-traumatic BME. In this study, we present data for pathological causes other than trauma as etiologies for BME.

## Materials and methods

The study is an observational, descriptive, and cross sectional study of autopsy cases.

The cases were examined in the central forensic pathology laboratory in Cairo, Egypt.

This study protocol was reviewed by the Research Ethics Committee (REC) for Human and Animal Research at the Faculty of Medicine at Helwan University (serial no. 21–2021).

### Case selection

Cases referred to the central forensic pathology lab in Cairo for a period of 2 years, with identifiable BME in the pulmonary vessels, were selected, regardless of the circumstances or the cause of death.

Eleven cases are selected from 400 consecutive autopsy cases based on the presence of BME or fat embolism. Anonymous archived data (no personal information) were used in this study.

Since the data was archived, no consent to participation and publication forms were signed nor collected.

### Autopsy and histopathological study

Routine autopsies included complete dissection for macroscopic evaluation of organs, including the heart, lungs, and brain. The kidneys, liver, and gastrointestinal organs, if relevant, were also examined.

BME is detected morphologically by the routine H&E stain (fat and immature blood elements including megakaryocytes in the pulmonary vessels point to bone marrow embolism). Martius scarlet blue trichrome (MSB) stain is used to highlight fibrin. CD117 immunostains were used to highlight the hematopoietic progenitor cells. Photos were taken by AxioCam ERc5s Zeiss camera connected to Zeiss microscopy.

## Results

Clinical data, if any, autopsy, and pathology diagnoses are grouped in the following table (Table 1).

**Table 1:** Characteristics, autopsy findings, and histopathological findings of BME cases

**#**	**Circumstances**	**Gross autopsy findings**	**Organs**	**Microscopic findings**	**Cause of death**
Case 1	Male in his 50s Death following struggle and trauma	Facial injury and an arm fracture	Lungs Heart	Pulmonary embolism (fibrin and bone marrow) associated with severe congestion, hemorrhages, and focal atelectasis-features of shock Cardiomegaly, mild coronary stenosis	Shock lung
Case 2	Male in his 40s Died following a struggle	Multiple fractures	Lungs Heart	Pulmonary fibrin thrombi, with BME, bone embolus associated with features of shock Unremarkable	Shock lung
Case 3	Female in her 30s Death following trauma. Shocked with hyper viscosity	Multiple traumas, and fractures	Heart Lungs	Heart congestion Shock lung, fat embolism	Fat and bone marrow embolism
Case 4	Male in his 50s Death during renal surgery	Congested facies A sutured lateral abdominal incision. Renal sutures and ureteric stent	Brain Lungs Kidney	Severe brain congestion Shock lung and BME Mild hydronephrosis and mild pelvic inflammation	Shock lung BME
Case 5	Female in her 30s Positive history of drug addiction (tablets)	Found dead, no signs of trauma	Heart Lungs	Few macrophages, minimal granulation in the myocardium Acute lung injury and BME	Acute lung injury
Case 6	Female in her 30s Postpartam hemorrhage.	Post-CS hemorrhagic shock with disseminated intravascular coagulation	Heart Uterus Lungs Brain	Congestion Suture site inflammation. Bilateral broad ligament hematomas exaggerated placental site change Shock lung with BME Severe congestion	DIC
Case 7	Male in his 50s with history of respiratory distress and no signs of trauma	No signs of trauma	Lungs Heart Brain	Pulmonary fibrosis with interstitial inflammation, mild eosinophilia, mild bronchitis, and grade III pulmonary hypertension with *Aspergillus* fungal balls, and BME Cardiomegaly with concentric hypertrophy. Coronary atherosclerosis with mild left anterior descending stenosis and right old recanalized thrombus Features of ischemic heart disease Mild mitral and tricuspid thickening Congested Brain with lacunar infarcts (hypertensive changes)	Ischemic and hypertensive heart disease complicated by advanced pulmonary hypertension
Case 8	A male with history of dyspnea	No signs of trauma with cardiomegaly	Heart Lungs	Cardiomegaly 587 gm with right ventricular dilatation. Atherosclerosis with calcifications, no stenosis. Minimal myocardial fibrosis Grade IV pulmonary hypertension, BME	Right-side heart failure Advanced pulmonary hypertension
Case 9	Male is his 70s Hospitalized and died after 1 day with history of heart and lung diseases	No signs of trauma	Lungs Brain Heart Liver Kidneys	Mucinous carcinoma, BME, bronchitis, emphysema with advanced pulmonary hypertension, perivascular giant cell reaction Meningitis Arteriolosclerosis Chronic hepatitis, mild activity, (HAI 8/18, stage 3/6), 10% steatosis Atherosclerosis of the renal artery, arteriolosclerosis Renal artery atherosclerosis with mild stenosis, renal arteriolosclerosis, and renal pyemic abscesses	Cancer
Case 10	Male in his 60s with jaundice due to hepatic malignancy.	Death in custody	Heart liver Lungs	Advanced atherosclerosis with mild right coronary stenosis and minimal septal interstitial hemorrhage. A microfocus of lymphocytic aggregate in the pericardium. Right ventricular luminal marrow Hepatocellular carcinoma (HCC) complicating liver cirrhosis BME	HCC
Case 11	Female in her 50s	Death during liposuction	Heart Lungs	Unremarkable heart Pulmonary edema, shock lung with fat embolism	Shock lung

### Cases (1, 2, and 3) “Traumatic”

**Fig. 1:**
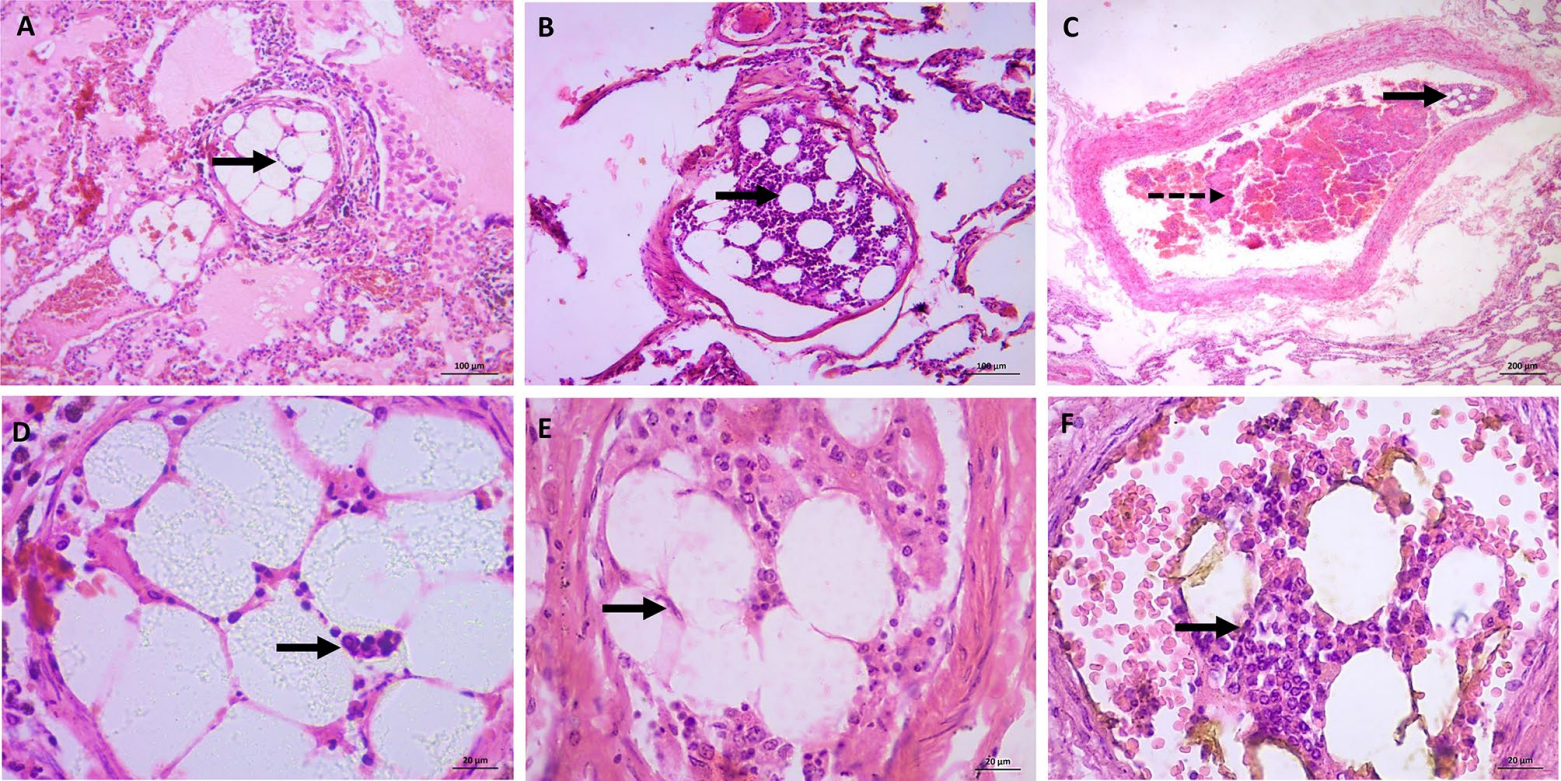
H&E-stained slides for cases 1–2-3: death following trauma and pulmonary embolism (bone marrow and fibrin). **A** and **B** (X40) medium size pulmonary vessel with bone marrow elements including hematopoietic cells and fat cells (thick arrows). **C** (X100) medium size pulmonary vessel with bone marrow elements (hematopoietic cells and fat cells) and fibrinous matrix entangling mixed blood elements denoting recent thrombus (dashed arrow). **D**, **E**, and **F** (X400) medium size pulmonary vessel with bone marrow elements (hematopoietic cells and fat cells) (thick arrows)

Cases 1, 2, and 3 showing BME following trauma are illustrated in Fig. 1.

Case 1, a male in his 50s, displayed facial injuries and arm fractures on gross examination. The histopathological examination of the lungs revealed a pulmonary BME with fibrin thrombi in medium sized vessels, highlighted by MSB stain (Fig. 5A, B). Patient history suggested that fractures pushed — by increasing intramedullary pressure — the bone marrow to enter the venous circulation and reached the lungs, causing serious respiratory insufficiency. Salient findings were severe edema, hemorrhage, and collapsed alveoli, the classic features of shock lung. Histopathological examination of the heart revealed mild coronary stenosis and cardiomegaly.

Case 2, a male in his 40s, showed multiple fractures in gross examination. A pulmonary BME was noted during histopathological examination of the lungs. The embolus was accompanied by features suggestive of shock lung: severe congestion, hemorrhage, and focal collapsed alveoli.

Case 3, a female in her 30s, displayed multiple trauma and fractures. Heart examination was unremarkable showing only congestion. She suffered from blood hyperviscosity syndrome. She exhibited shock lung as her lungs were not filled with sufficient air. The body organs did not receive enough oxygen for normal functions. This patient developed fat embolism that occurred when embolic fat macroglobules passed into small vessels of the lungs and other organs. Autopsy also showed some minimal marrow elements.

### Cases (4, 5, 6, 7, and 8) “Non-traumatic”

Cases 4 and 5 presented with acute lung injury as the cause of death.

**Fig. 2:**
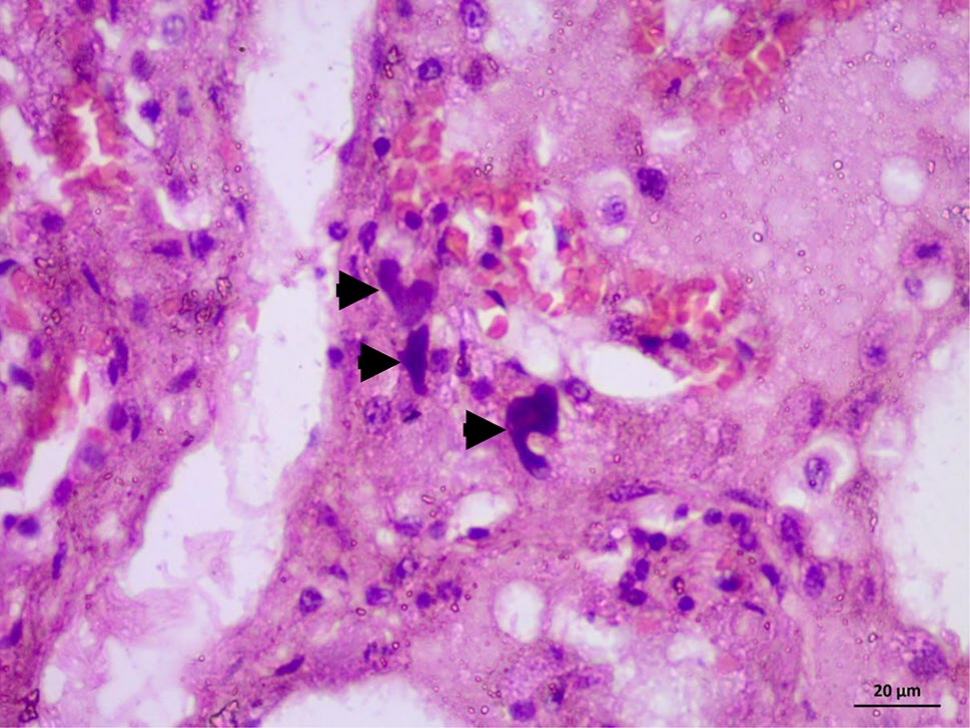
H&E-stained slides. (X400) for case 4: death during renal surgery, BME, and shock lung. Photo showing severe congestion, extravasation, edema, early neutrophilic, and entrapped megakaryocytes (arrow heads), microscopic features of shock lung

Case 4, a male in his 50s, died during renal surgery due to a complication of anesthesia. He exhibited facial congestion. A sutured lateral abdominal incision was observed. Renal sutures and a ureteric stent were also reported on gross examination. Histopathological examination of the brain revealed severe congestion. Figure 2 shows severe congestion, extravasation, edema, early neutrophilic, and entrapped megakaryocytes (Fig. 2), microscopic features of shock lung.

**Fig. 3:**
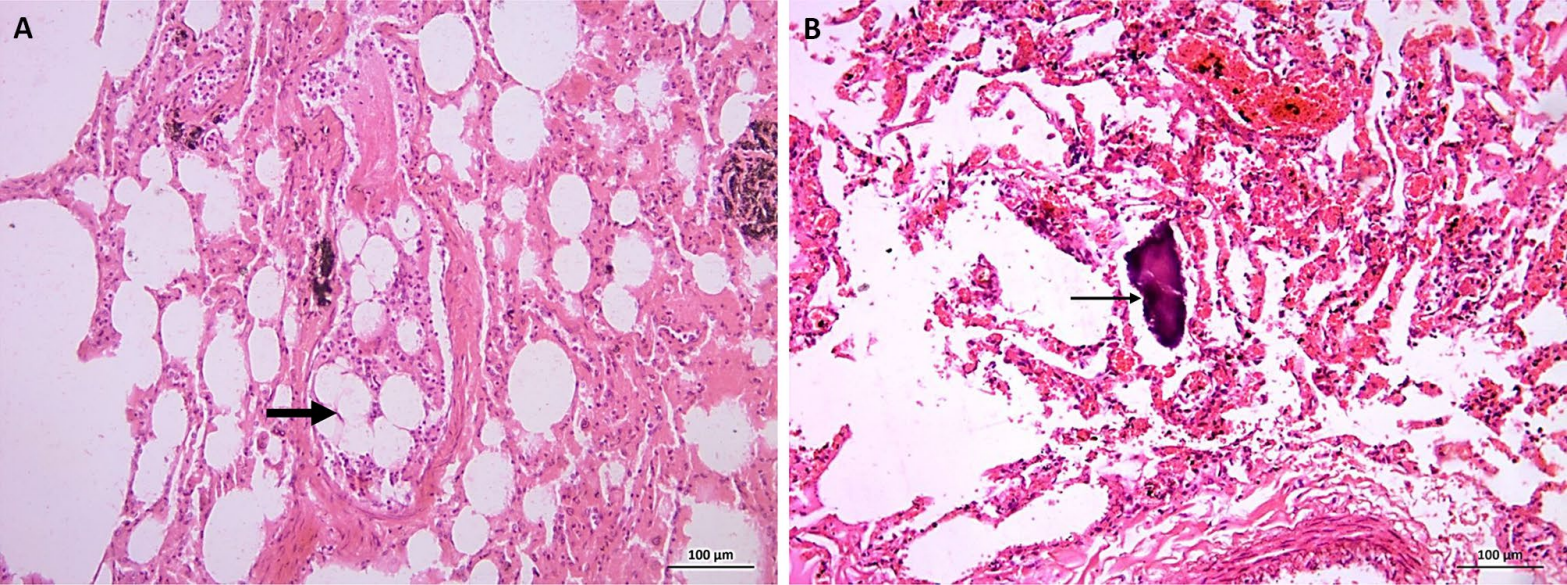
H&E-stained slides for case 5: positive history of drug addiction and death of acute lung injury. **A** (X40) medium size pulmonary vessel with bone marrow elements (hematopoietic cells and fat cells) (thick arrow). **B** (X40) bony fragment denoting filler lung (thin arrow)

Case 5 is a female with a history of drug abuse. Histopathological examination of the heart showed few macrophage and micro foci of granulation tissue in the myocardium. The coronaries were patent. However, drug intake can cause the respiratory centers to depress, resulting in hypoxic episodes (Fig. 3).

**Fig. 4:**
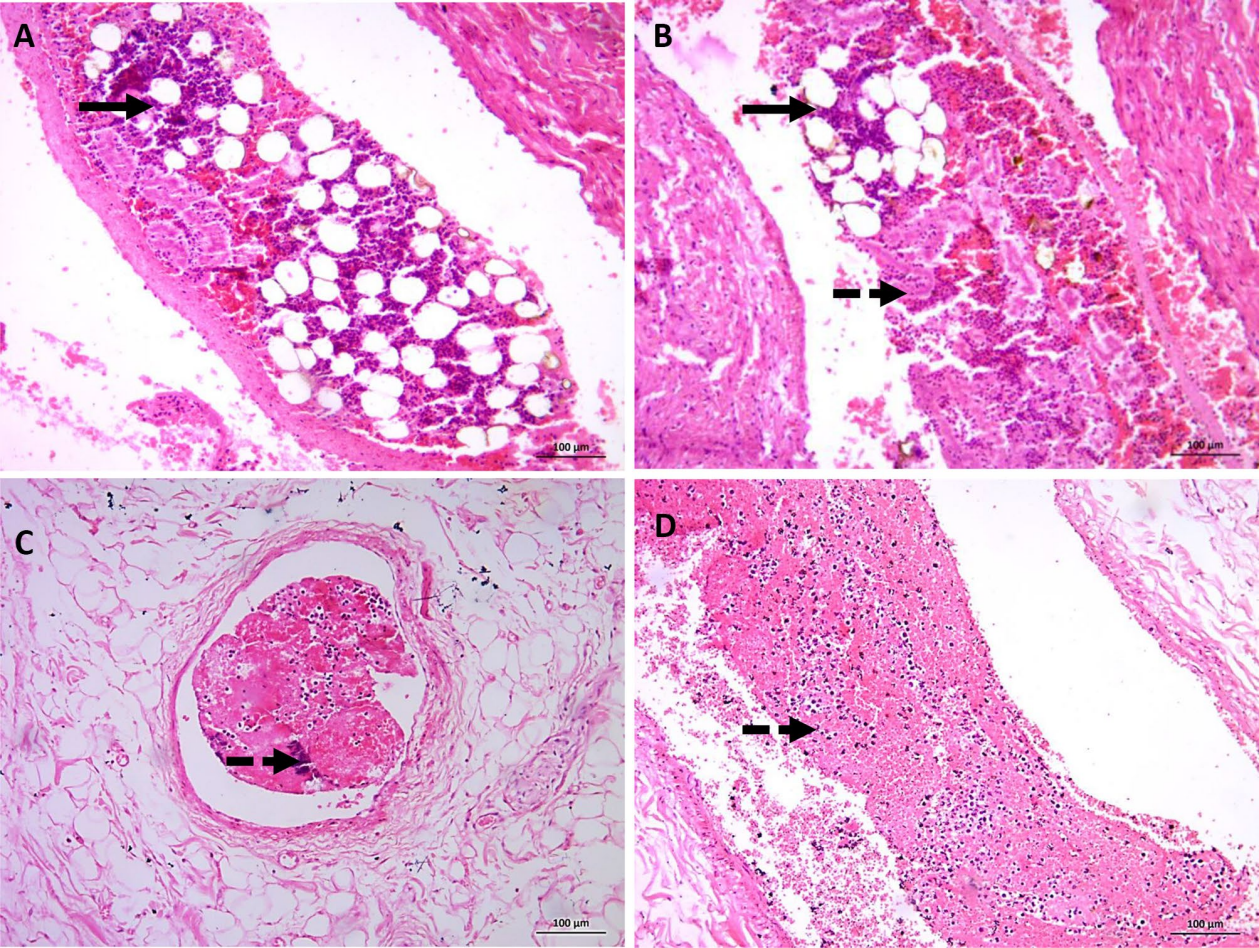
H&E-stained slides for case 6: post-CS hemorrhagic shock and DIC. **A** and **B** (X40) large size pulmonary vessel with bone marrow elements (hematopoietic cells and fat cells) (thick arrows) with fibrin thrombi dashed arrow in **B**. **C** and **D** (X40) fibrin thrombi (dashed arrows)

**Fig. 5:**
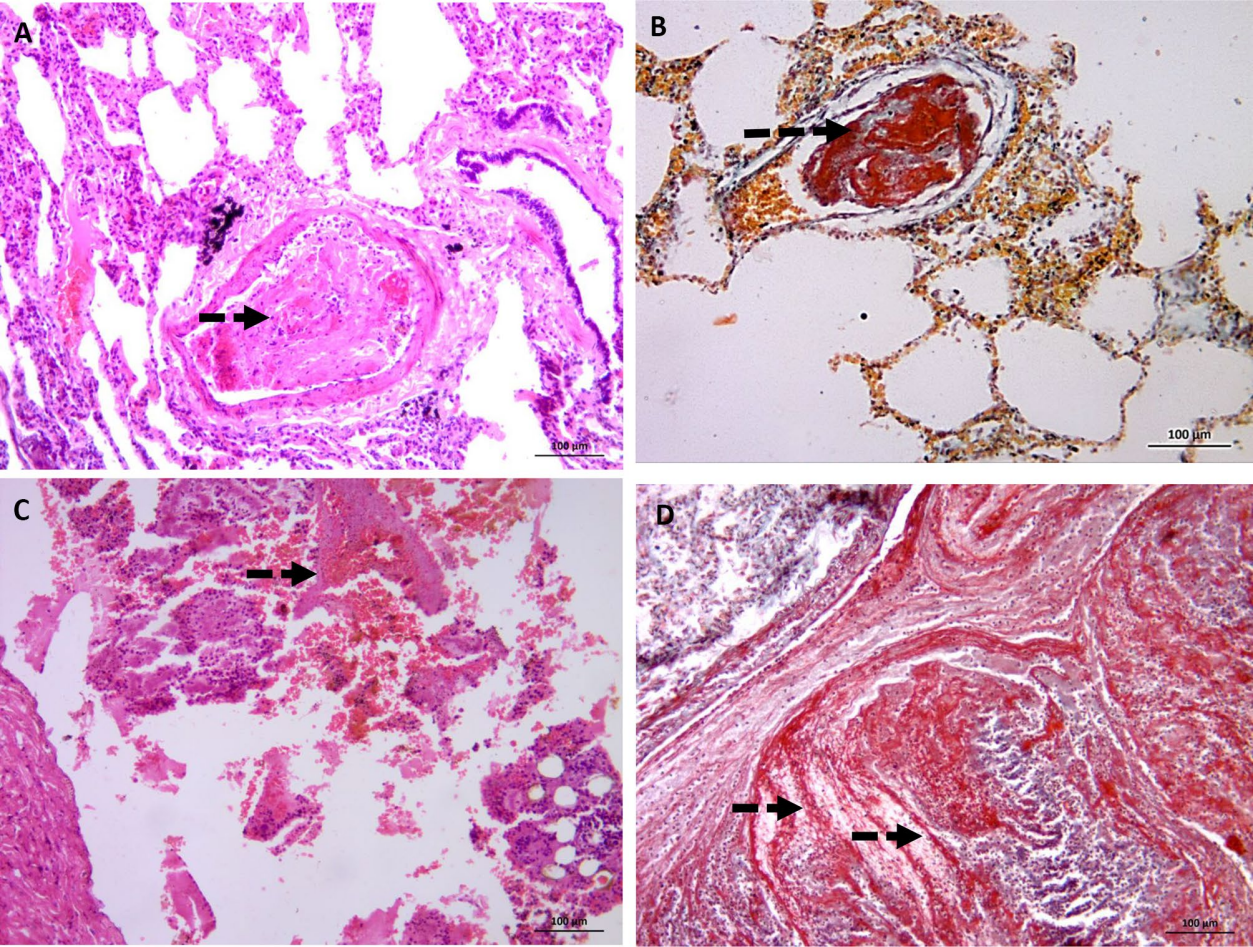
For cases 1 and 6: H&E-stained slides. **A** and **C** (X40) fibrin thrombi with lines of Zahn (dashed arrows). MSB-stained slides. **B** and **D** (X40) fibrin thrombi with lines of Zahn (dashed arrows)

Case 6 is a female in her 30s who suffered from hypovolemic shock due to post-CS hemorrhage and DIC. Any sustained shock, following severe injury and hemorrhage, stimulates bone marrow function and hematopoietic stem/progenitor cell proliferation and differentiation. These processes could lead to BME (Fig. 4). Thrombi are present in a large size vessel as highlighted (Fig. 5C, D).

**Fig. 6:**
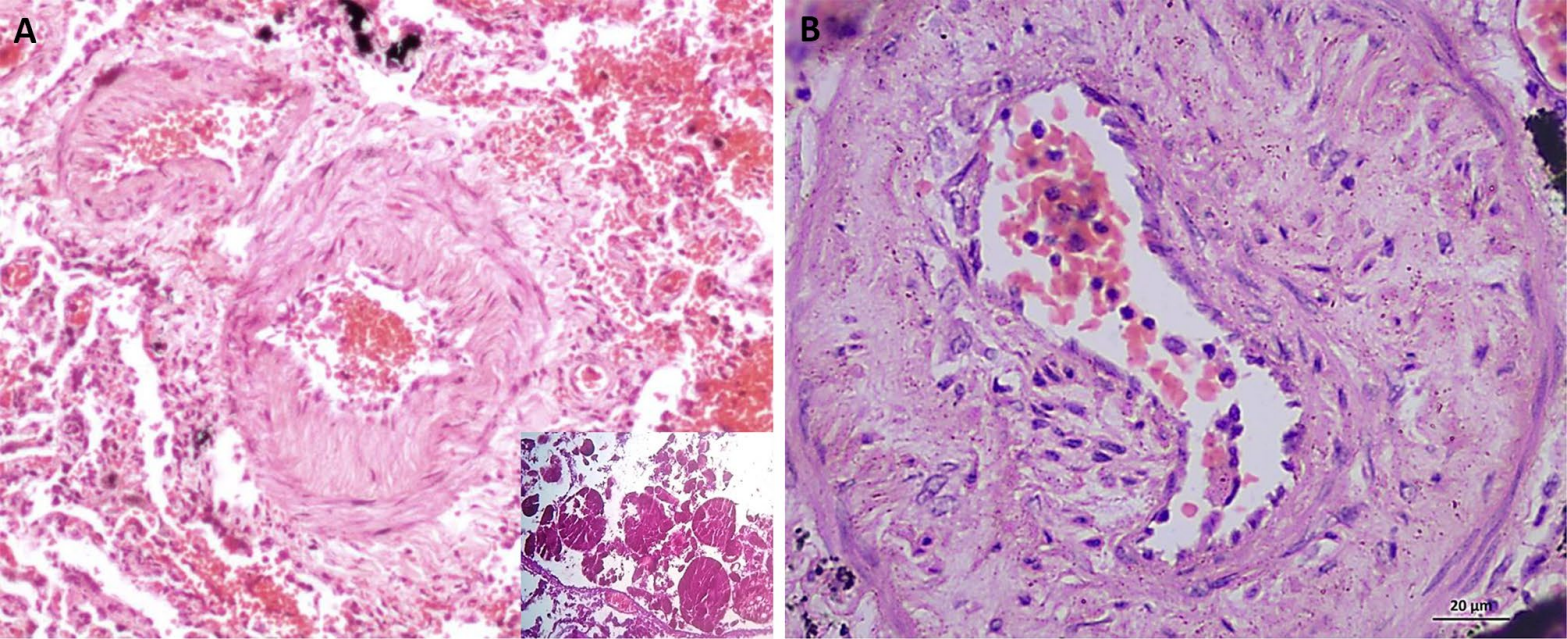
H&E-stained slides for case 7: respiratory distress. **A** (X100) severe congestion, extravasation, edema, and early neutrophilic, microscopic features of shock lung. Grade III pulmonary hypertension with markedly thickened pulmonary vessels. Aspergillus fungal balls (insert lower left). **B** (X400) high power view of pulmonary vessel with medial hypertrophy and intimal fibrosis

Cases 7 and 8 are individuals with shock and advanced pulmonary hypertension. Case 7 is a male in his 50s with a history of respiratory distress but no signs of trauma. Histopathological examination of the lung revealed pulmonary fibrosis, grade III pulmonary hypertension, non-invasive *Aspergillus* fungal balls, and BME (Fig. 6).

**Fig. 7:**
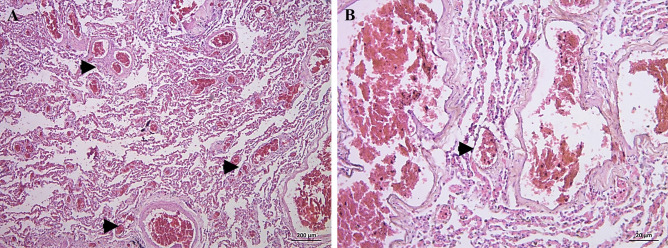
H&E-stained slides for case 8: right-sided heart failure associated with advanced pulmonary hypertension. **A** (X100) severe congestion with extravasation. Plexiform lesion characteristic of grade IV pulmonary hypertension (arrow heads). **B** (X400) high power view of pulmonary vessels with ectasia and sprouting of branching plexiform capillaries (arrow head)

Case 8 is a male with a history of dyspnea but no signs of trauma. He had cardiomegaly (587 gm) with right ventricular dilatation (right-side heart failure) and grade IV pulmonary hypertension. Histopathological examination of the heart tissue revealed advanced atherosclerosis with calcification; however, stenosis was unremarkable. Minimal myocardial fibrosis was noted. A decline in heart-pumping capacity led to volume overload in the left ventricle resulted in over-abundance of blood in the left atrium causing backward pressure on the lungs. The result was pulmonary hypertension. This condition, over time, produced right ventricle dilatation and cardiomegaly (Fig. 7).

### Cases 9 and 10 (cancer)

Case 9 is a 70-year-old man who died of cancer. Autopsy revealed mucinous carcinoma, bronchitis, emphysema, advanced pulmonary hypertension, perivascular giant cell reaction, and BME in the lung. Gross autopsy and radiology findings did not suggest any fractures. Autopsy of the liver, brain, heart, and kidneys revealed chronic active hepatitis, atherosclerosis, and arteriolosclerosis. Autopsy of the brain and kidneys revealed meningitis and renal pyemic abscesses. This constellation of findings is most likely a consequence of cancer. Chronic hepatitis was part of this constellation.

Case 10 is a 60-year-old male with jaundice; there were no gross findings in the autopsy. Autopsy of the heart revealed advanced atherosclerosis, stenosis, and inflammation along with right ventricular marrow, indicating extramedullary hematopoiesis. Liver autopsy revealed hepatocellular carcinoma, complicated by cirrhosis; this finding may explain jaundice and atherosclerosis.

### Case 11 (liposuction)

**Fig. 8:**
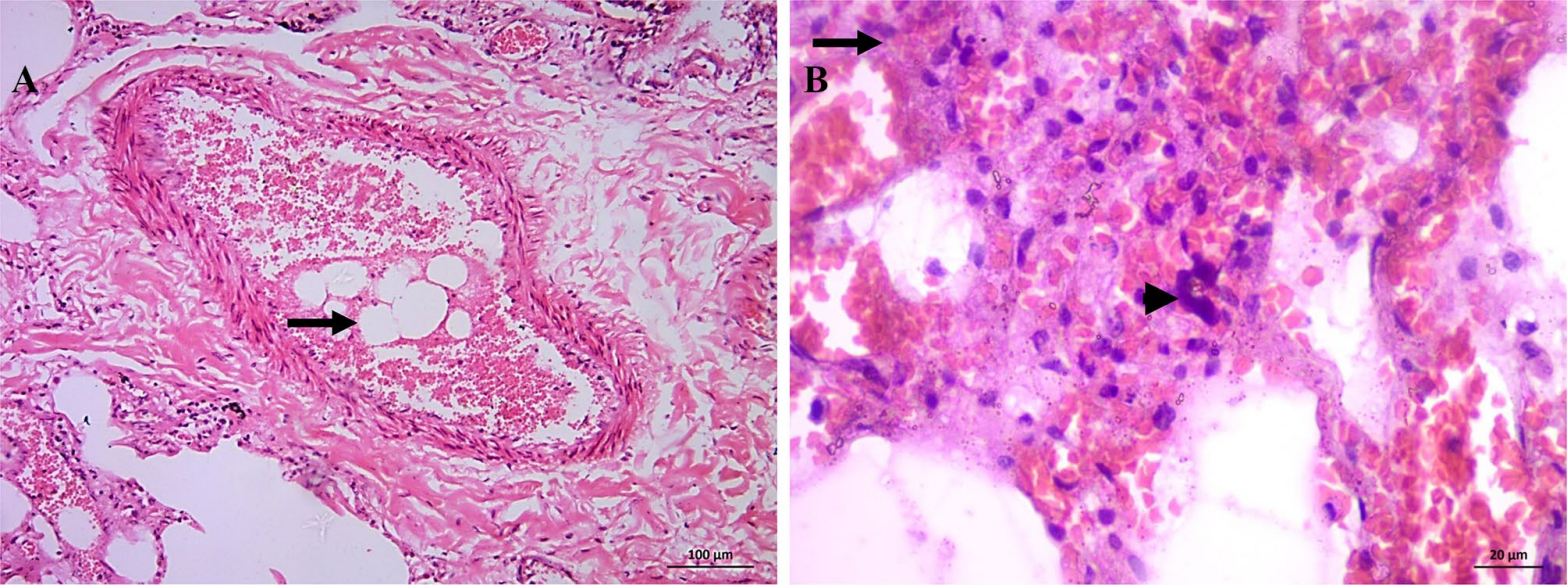
H&E-stained slides for case 11: liposuction. **A** (X100) medium size pulmonary artery with fat emboli (thick arrow). **B** (X400) severe congestion, extravasation, edema, early neutrophilic, and entrapped megakaryocytes (arrowhead). Microscopic features of shock lung

A female in her 50s died during liposuction. She suffered from shock lung with fat embolism and pulmonary edema (Fig. 8).

**Fig. 9:**
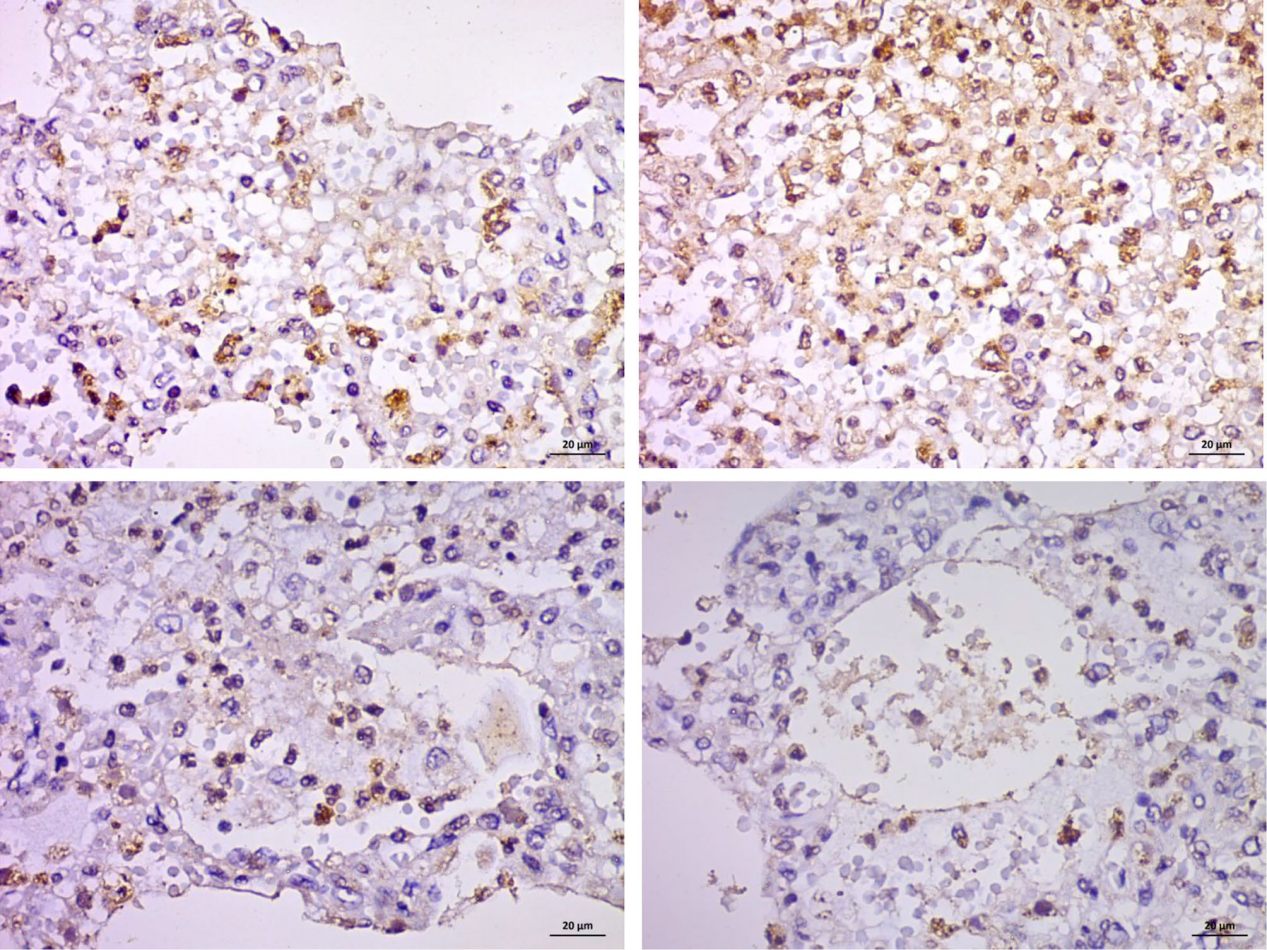
CD117-immunostained slides, (X400) the CD117 highlights the immature marrow elements in the capillaries

As shown in Fig. 9, the CD117 highlights the immature hemopoietic cells and immature marrow elements in the capillaries.

## Discussion

The cause of death in the previous described cases 1, 2, and 3 is BME and shock lung after a quarrel and consequent trauma. These findings are consistent with the common concept in the literature that BME occurs after multiple trauma and bone fractures. For case 4, the histopathology of the lungs revealed BME. Cardiovascular collapse or the state of shock seems to have a direct correlation with the numbers of megakaryocytes in the blood. These cells could be a factor in BME [30]. The presence of bone marrow in pulmonary vessels can increase vascular permeability to proteins, thereby increasing pulmonary arterial pressure and causing pulmonary edema [31]. Activation of the coagulation cascade may also occur, which, in turn, increases activation of platelets and their release into the pulmonary circulation. This release worsens pulmonary edema. Finally, shock lung develops, which was the cause of death for this individual [32]. Hematopoietic stem cells (HSC) are located in the stroma of the bone marrow. In the presence of the relevent stimuli, they produce huge, diverse colonies of mature functional blood cells. Then the maturing cells travel from the bone marrow to the peripheral blood where they replace malfunctioned cells and maintain immune function. Furthermore, HSC differentiate into multipotent progenitor cells that become lineage-restricted during proliferation and maturation. However, small numbers of immature progenitor cells pass into the periphery to aid in the repair. HSC and hematopoietic progenitor cells are not found in the peripheral circulation under normal conditions [33].

Myocardial granulation tissue in case 5 is a likely a consequence of hypoxia and respiratory center depression caused by drug abuse. Death probably occurred due to an overdose that caused marked respiratory suppression and shock lung with BME. A less likely hypothesis is a relationship between drug abuse and septic inflammation, such as osteomyelitis. Thus, BME could be attributed to osteomyelitis, which would reduce bone marrow integrity [34]. Embolism leads to an increase in pulmonary vascular permeability to proteins. This increase elevates pulmonary pressure and finally causes pulmonary edema. Edema is aggravated by an increased tendency of megakaryocytes to deposit in the lungs. Karyocyte deposition and the resultant platelet activation can eventually cause acute lung injury and death [35].

For case 6, hypovolemic shock leads to disseminated intravascular coagulation (DIC) associated with increased maternal morbidity and mortality. DIC produces widespread microvascular thrombosis, which can compromise the blood supply and cause various organs to fail. Finally, exhaustion of coagulation/anticoagulation factors and platelets may lead to profuse uncontrollable bleeding and, often, death [36]. In fact, during normal pregnancy, a prothrombotic state is more active than fibrinolysis (hyper state of coagulation). This response is a natural protection against blood loss during and after delivery [37]. Two separate factors are postulated to induce DIC — slow capillary flow and secretion of thromboplastin into the blood. Experiments tend to confirm the hypothesis that a thromboplastic substance in the bloodstream is harmless when blood flow is normal. However, slowing of the capillary blood flow may cause the same amount of thromboplastic material, to produce DIC and cause death linked to clotting defect [32].

For case 7, non-invasive aspergillosis causes an allergic reaction and explains the histological finding of eosinophilia. BME may have been caused by cardiorespiratory failure. Furthermore, concentric hypertrophic foci to hypertensive heart disease affected cerebral vessels causing lacunar infarcts. Coronary atherosclerosis with ischemic heart disease aggravated the cardiac condition. These two conditions contribute to left- and right-side heart failure. Right-side failure leads to pulmonary hypertension that may result in cardiorespiratory failure.

The patient in case 8 underwent cardiogenic shock, which occurs when the heart cannot efficiently pump blood and oxygen. This shock led to a change in pressure in the fat-containing cavity of the bone marrow that allowed the escape of marrow elements and fat to the circulation. This hypothesis would explain the presence of BME without noticeable trauma [38].

Cardiovascular diseases are associated with bad prognosis in patients with malignancies. Patients with cancer, as in case 9, are often found to have conditions related to metabolic and vascular  pathologies, including abdominal obesity, altered glucose metabolism, lipoprotein abnormalities, and hypertension [39]. The chemical theory explains BME as a process that begins with lipoprotein lipase action on fat globules; then C-reactive protein and free fatty acids are released. These metabolites cause local and systemic inflammatory responses and may lead to direct injury by agglutination and vascular obstruction. Free fatty acids and other mediators are associated with inflammatory responses in the lungs, such as pneumonitis and vasculitis. This pathway for inflammatory response is thought to mimic the acute lung injury (ALI) and adult respiratory distress syndrome (ARDS) pathways. A study in rats with corn oil-induced fat embolism syndrome (FES), indicated markers of inflammation and microvascular obstruction, and increased permeability and pulmonary hypertension. They identified inflammatory cytokines, phospholipase A2, nitric oxide, and inducible nitric oxide synthase as the toxic biochemical mediators underlying the development of this condition [39].

An alternative explanation may be that cancer can weaken the immune system by spreading into the bone marrow. Lung cancer is a solid tumor with low antigenicity and a heterogenic phenotype that evades host immune defenses [40]. This cancer can lead to osteomyelitis that affects the bone marrow integrity and causes BME. These findings indicate that BME is not exclusively related to fracture or trauma [34]. As for case 10, concomitant ischemic heart disease and neoplasia in the same patient is not a rare occurance, and 4 to 10% of cases with acute coronary syndrome (ACS) or chronic ischemic heart disease have a history of cancer [41]. Chronic activation of the immune system and inflammatory state underlie the pathophysiology of atherosclerosis and neoplasms [41]. This concept would explain the finding of BME; the biochemical theory indicated that the clinical presentation of FES is inferible to a proinflammatory state. Bone marrow fat is catabolized by tissue lipases, resulting in increased levels of glycerol and toxic free radicals. These intermediary products lead to end-organ dysfunction. Toxic injury to pneumocytes and pulmonary endothelial cells induces vasogenic edema, cytotoxicity, and hemorrhage. Disrupted pulmonary endothelium triggers the cascade of proinflammatory cytokines and the progression to acute lung injury or acute respiratory distress syndrome [39].

During liposuction and fat grafting as in case 11, small blood vessels are ruptured, and the adipocytes are damaged, and consequently the lung injury is caused by the production of  lipid micro fragments reaching the venous circulation. Liposuction-induced fat embolism syndrome classically occurs 12 to 72 hours after surgery.

Three theories are reported to describe the pathogenesis and the timing of the embolic events of this syndrome; first, the mechanical theory suggets that fat cell disruption in the fractured bone leads to the release of fat droplets. Fat droplets enter the torn veins near the injury and are then transported to the pulmonary vascular bed. Large fat globules form in this region and result in mechanical obstruction when trapped in the lung capillaries. Still, this theory does not provide explanation for cases showing delayed onset of symptoms (over 72 hours) following liposuction [42, 43].

An alternative biochemical theory explains non-traumatic and delayed fat embolic events. This theory postulates that when fat globules reach the pulmonary capillaries, pneumocytes produce hydrolytic lipase which convert fats into glycerol and free radicals. High concentrations of these toxic byproducts trigger alveolar and endothelial cell injury. This injury inactivates lung surfactant release due to type II pneumocyte apoptosis. Finally, vascular permeability increases via the release of vasoactive amines and prostaglandins and recruitment of neutrophils. These alterations induce interstitial and alveolar hemorrhage, edema, chemical pneumonitis, and formation of hyaline membrane. This multi-step process of fat degradation suggested by the biochemical theory proposes an acceptable explanation to the delayed onset of symptoms related to embolism following liposuction. A local inflammatory process is also required before the symptoms appear. Additional evidence of this theory is reported in cases with non-traumatic aetiology, such as inflammation in pancreatitis. Serum from acutely ill patients can induce agglutination of chylomicrons, low-density lipoproteins, and liposomes of nutritional fat emulsions. In such patients, the levels of C-reactive protein are elevated, indicating the ability to induce calcium-dependent lipid agglutination [42, 43].

The third and most recent theory is the least supported. It is the coagulation theory suggesting the release of tissue thromboplastin and marrow elements after long bone fractures, followed by triggering the complement system and the extrinsic coagulation cascade. These events lead to intravascular coagulation via fibrin and fibrin degradation products, which combine with leukocytes, fat globules, and platelets to increase pulmonary vascular permeability. Permeability increases through direct action on endothelial cells and indirectly through the release of vasoactive substances. However, this theory fails to validate the etiology of non-traumatic FES. These three theories may coexist, and are not necessarily mutually exclusive. They have all been reported after major traumatic events involving long bone fractures, and following intramedullary orthopedic procedures. These theories likely play a contributory role to the etiology and time path of traumatic versus non-traumatic pathogenesis of FES) [44].

## Conclusion

BME, which is rarely observed in man, is attributed to traumatic and non-traumatic causes; two theories describe the mechanism of BME: chemical and mechanical.

The chemical theory explains BME in non-traumatic cases as attributable to a proinflammatory state. In traumatic cases, the mechanical theory explains BME as a consequence of a transient increase in the pressure of fat-containing cavity in association with torn blood vessels. This condition allows the marrow and adipose fat cells to escape into the circulation. The autopsy findings in the 11 cases discussed above contradict the common concept of BME as exclusively a consequence of traumatic injury.

BME is a lesion in which the bone marrow elements, including cell debris and yellow bone marrow, reach the systemic circulation and invade the lung parenchyma through the venous sinuses. Non-traumatic cases of BME observed in individuals with cancer, atherosclerosis, DIC, and drug abuse can be explained by the biochemical theory in which the clinical presentation of FES is inferible to a proinflammatory state. As most legal authorities may view BME as a signal of traumatic death, which in turn may have additional legal consequences especially in solving cases of homicides and violence, the identification of postmortem findings of BME owing to non-traumatic causes is of major relevance in the forensic pathology discipline.

## Key points

1. Autopsy cases show evidence that BME may be caused by non-traumatic injuries.
2. The pathophysiology behind non-traumatic BME is still unclear and further studies to be fully understood.
3. Cardiovascular collapse or the state of shock seems to have a direct correlation with the numbers of megakaryocytes in the blood and in turn related to non-traumatic BME.
4. Over-dose intoxication may be related to BME through hypoxia and cardiorespiratory center failure.

## Data Availability

The datasets used and/or analyzed during the current study are available from the corresponding author on reasonable request.
